# Multiple Myeloma Mimicking a Small Vessel Vasculitis Presentation

**DOI:** 10.1155/2020/9146842

**Published:** 2020-02-12

**Authors:** Mateo Mejía-Zuluaga, Jorge Andrés Lacouture, Maria Clara Gaviria, Maria Adelaida Garcés, Ana María Mejía, Sebastián Herrera

**Affiliations:** ^1^Resident of Internal Medicine, CES University, Medellín, Colombia; ^2^Resident of Dermatology, CES University, Medellín, Colombia; ^3^Dermatology, Hospital General de Medellín, Medellín, Colombia; ^4^Rheumatologist, Hospital General de Medellín, Medellín, Colombia

## Abstract

Multiple myeloma can have different clinical manifestations, and not all patients present with classic CRAB component. We describe a 46-year-old woman admitted to our hospital with a complaint of a bluish-to-black discoloration of the second toe that was rapidly progressive and acute kidney injury. We documented a Kappa light chain monoclonal gammopathy, increased presence of plasmacytes in bone marrow aspiration, and multiple lytic bone lesions, which led to a diagnosis of multiple myeloma. Although multiple myeloma presenting with blue finger syndrome is uncommon, it must always be considered as a differential diagnosis with this clinical finding.

## 1. Introduction

Multiple myeloma can have different clinical manifestations, and not all patients present with a classical CRAB component: anemia, hypercalcemia, osteolytic lesions, and kidney injury [1].

Kidney involvement is a usual characteristic of multiple myeloma in association with anemia or hypercalcemia and with evidence of mono (majority of cases) or polyclonal gammopathy in protein electrophoresis [2]. The presentation of a blue digit as a manifestation of multiple myeloma is uncommon. Usually vasculitis is the underlying disease [3].

We describe the case of a patient who was admitted because of a blue finger syndrome that was widely studied, until determining that her causative disease was multiple myeloma.

## 2. Case Report

**Figure 1:**
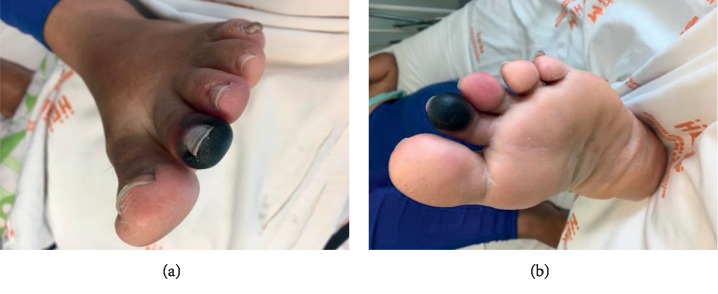
Black coloration (necrosis) of the second left-foot finger at admission.

A 46-year-old Hispanic woman was admitted to our hospital with a chief complaint of a bluish to black coloration of her second toe (Figure 1) associated with pain that started 3 days prior to admission. The symptoms were preceded by 2 months of intermittent claudication in the affected limb.

The patient also described arthralgia involving proximal interphalangic joints, shoulders, elbows, and knees, with morning stiffness and foamy urine. She denied recent trauma to the affected limb. Her past medical history was relevant for arterial hypertension, hypertriglyceridemia, and aortoiliac atherosclerosis. Her medications were amlodipine, captopril, hydrochlorothiazide, and gemfibrozil.

Physical examination revealed necrosis of the second toe of her left foot, with tenderness and reduced capillary refill. Pulses were palpable over the pedal arteries in both limbs. Skin examination showed livedo reticularis and scant brown macules over her thighs. No ulcers were identified. The rest of the physical examination was normal.

The initial laboratory tests were hemoglobin 12 (gr/dL), hematocrit 37%, leukocytes 10,200 (mm^3^) [3], neutrophils 76%, platelets 564,000 (mm^3^), creatinine 1.64 (mg/dL), BUN 25 (mg/dL), glycaemia 121 (mg/dL), PT 14.7, INR 1.03, PTT 29/28.5, ESR 2 mm/h, and CRP 1.2 mg/dL. Urine test: proteinuria +++ estimated in 300 mg/dL, RBC 6/hpf, WBC 4/hpf, and RBC casts: present.

**Figure 2:**
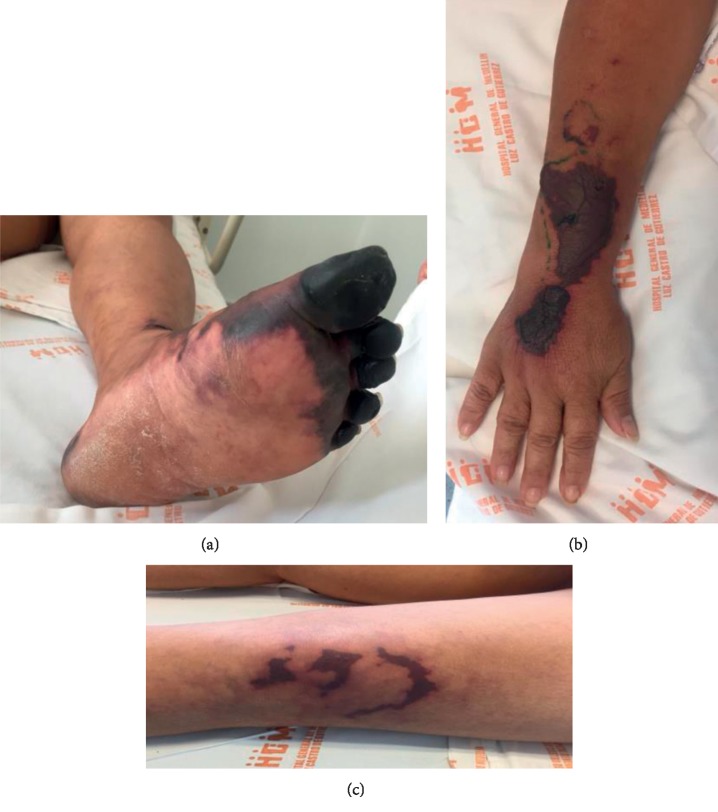
Progression of necrotic lesions on the left foot: new lesions involving the right arm skin and retiform purpura.

Given her past medial history and physical examination, peripheral artery disease was the first clinical diagnosis. However, results of the arterial and venous Doppler ultrasonography showed permeable vascular beds. At this point, a systematic approach to common etiologies of blue-finger syndrome was undertaken. We sought out embolic etiologies including microthrombi of cardiac origin and cholesterol emboli, hypercoagulable states such as antiphospholipid syndrome and autoimmune diseases. Emphasis was made on vasculitis given the glomerular involvement showed in urinalysis results and decreased glomerular filtration rate. Test results of this approach are shown in Table 1. At the time the results were obtained, the patient's necrosis and skin lesions worsened (Figure 2).

Due to the worsening condition in a patient with digital ischemia and suspected glomerular disease, vasculitis was considered as one of the possible etiologies, and empirical immunosuppression was started with methylprednisolone pulses. Renal and skin biopsies were ordered, and protein electrophoresis was also obtained to rule out less-frequent causes of hypercoagulable states and kidney failure such as monoclonal gammopathies.

**Figure 3:**
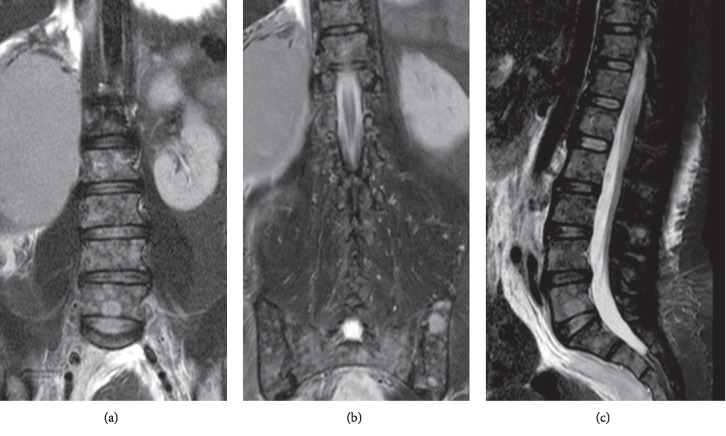
Magnetic resonance images enhanced in short inversion time inversion recovery (STIR) in coronal (left and middle) and sagittal (right). The alteration in the signal of the bone marrow of the vertebral bodies and both iliac bones can be seen by countless discrete images with hyperintense signals of contours, rounded, diameters of up to 13 mm, which are compatible diffuse commitment by multiple myeloma. In addition, the asymmetry in the size of both kidneys can be observed. The adequate attention in a lesion suggestive of neoplasia.

Serum protein electrophoresis did not show monoclonal spikes or any other abnormality, and, despite immunosuppressive therapy, her condition worsened with progressive kidney injury requiring renal replacement therapy with hemodialysis. The patient developed pain in the lumbar region with associated tenderness during treatment, which was interpreted as a possible infection unmasked by the immunosuppression therapy, and a magnetic resonance image of the lumbar column was performed showing an unexpected result (Figure 3).

The radiology report of the magnetic resonance image of the lumbar column described polyostotic lytic lesions of the axial skeleton and pelvic structures compatible with neoplastic lesions (Figure 3). We also received skin biopsy results showing residual inflammatory finding compatible with a resolving vascular process with no signs of cholesterol emboli and renal biopsy findings of cast nephropathy compatible with multiple myeloma. Given the results previously mentioned, we ordered serum immunofixation that reported monoclonal gammopathy of Kappa light chains and urine immunofixation with Kappa biclonal gammopathy, bone marrow aspiration, and biopsy with increased presence of plasmacytes (20.09%) with kappa monoclonality.

Given the presence of monoclonal gammopathy of Kappa light chains, acute kidney injury, increased presence of plasmacytes in bone marrow aspirate, and multiple lytic bone lesions, the patient was given the diagnosis of multiple myeloma and was started on plasmapheresis and chemotherapy based on cyclophosphamide, bortezomib, and dexamethasone. After 3 sessions of plasmapheresis and 28 days of chemotherapy, the patient's acute kidney injury resolved, she no longer needed renal replacement therapy. Most of her skin lesions improved, a trans-metatarsal amputation of the left foot was required, and she was sent home to continue ambulatory treatment.

## 3. Discussion

Malignancy can sometimes be a challenging diagnosis because of the possibility of multiple symptoms and clinical manifestations. Despite a well-recognized triad in multiple myeloma (anemia, hypercalcemia, and acute kidney injury), it is not always present, and there should always be a heightened clinical suspicion.

Renal involvement as a first manifestation of the disease can be challenging due to multiple types of injury (prerenal, renal, postrenal) with a wide spectrum of differential diagnosis [1]. The etiology of each of the mechanisms involved in myeloma's kidney disease is worth mentioning:

- Prerenal: hypovolemia induced by hypercalcemia, gastrointestinal losses, or hyperviscosity.
- Renal (“myeloma kidney”): glomerular disease from amyloid or light chain deposition. Proximal tubular injury from light chains, uric acid, and casts (cylindruria).
- Postrenal: calculi and casts creating intrinsic obstruction.

Renal insufficiency is present in at least half of the myeloma patients and is associated with increased mortality. The three forms of renal injury (cast nephropathy, monoclonal immunoglobulin deposition disease, and light chain amyloidosis) can coexist, but cast nephropathy is the most prevalent [2, 3].

The finding of a blue finger in association with glomerular findings in the absence of anemia, hypercalcemia, and monoclonal gammopathy in the serum protein electrophoresis made systemic vasculitis as the first clinical possibility. Multiple myeloma was suspected when the result of the renal biopsy was known, which emphasizes the importance of both serum and urine protein electrophoresis and protein immunofixation, to avoid masking of the monoclonal peak (in blood) due to massive proteinuria. Our patient had 15 grams/day of urine protein.

Kidney involvement in multiple myeloma is a diagnostic challenge, and for this reason, multiple screening tests have been proposed, creating high sensitive and fast tests such as free serum light chain quantification and the Kappa–Lambda ratio [4]. Despite these novel tools, kidney biopsy remains the gold standard for diagnosis.

The patient received plasmapheresis initially because the most probable diagnosis was considered to be vasculitis (it is considered the standard treatment for small vessel vasculitis that required dialysis or had a serum creatinine over 5.8 mg/dl). With the diagnosis of cast nephropathy secondary to multiple myeloma, chemotherapy was initiated. There is also a possible clinical benefit removing free light chains by extracorporeal treatment [5–11]. For this reason, plasmapheresis was maintained until the initially planned 7 sessions were completed.

### 3.1. Differential Diagnosis

Blue digit syndrome is clinically seen as unilateral or bilateral purple or bluish discoloration of a finger or toe, due to ischemia. This entity may affect only one digit, but most frequently affects more than one. The affected digits are usually painful, and ischemia can evolve to ulceration, loss of tissue, infection, and irreversible necrosis with amputation [12].

Correct identification of the etiology of blue digit syndrome is of the outmost importance to define a proper management. There are multiple causes of blue digit syndrome and significant overlap in clinical presentation. As an initial approach, it is important to determine if the process is related to cold exposure or if its independent of temperature, as in the case of the patient. Skin biopsy is recommended if other signs of systemic disease are found [13].

Vasculitis was considered first as the most probable diagnosis given the multiorgan involvement (kidney, skin, and small vessels), and studies for ruling out secondary vasculitis and vasculitis mimics were undertaken. Deterioration of vascular lesions and renal function, despite immunosuppression, raised the possibility of a different diagnosis.

Cutaneous manifestations in paraneoplastic vasculitis are common findings and have been reported as the first clinical sign in approximately 1% of the cases [14]. Most of them are explained by vascular alterations, blood hypercoagulability in solid tumors, and secondary vasculitis (as small or medium size vessel) and erythromelalgia in hematological ones. Palpable purpura of the lower extremities is the main clinical feature of the paraneoplastic vasculitis, and it can occur a few years before any clinical manifestation of the tumor [14].

Even though cholesterol emboli syndrome can explain some of the signs and symptoms presented, it was excluded because the patient did not have the usual risk factors for them (no cardiovascular disease, no anticoagulation, no diabetes, no preceding cardiovascular intervention, no embolic sources identified in the echocardiogram, and no signs suggestive of cholesterol emboli in the skin or kidney biopsies (the pathology department was aware of this as a differential diagnosis in the presented case)).

In paraproteinemia causing hyperviscosity syndrome, the cutaneous findings are secondary to the stasis of blood (increased number of proteins or cells) and the vascular occlusion of the superficial vessels. Livedo reticularis, acrocyanosis and digital ischemia, which can progress to necrosis and gangrene, are common manifestations and undistinguishable from vasculitis [15]. Total serum proteins or gammaglobulin levels were not measured in this patient because the initial serum electrophoresis was normal. Free light chains Kappa and Lambda in serum were also normal: Kappa 137 mg/dl (normal value 170–370 mg/dl) and Lambda 56 mg/dl (normal value 90–210 mg/dl) with a normal K/L ratio of 2.43.

Monoclonal gammopathies of undetermined significance (MGUS), especially IgG/IgA type, have been associated with an increased risk of deep venous thromboembolism (DVT), arterial thrombosis, and coronary and cerebrovascular disease [16]. Anomalies in the environment of the stromal cells added to the effect of the monoclonal protein in formation, and an increase in factor VIII and von Willebrand factor could explain ischemic syndromes with or without skin manifestations [17].

Less than 2% of patients with MGUS have skin manifestations: angioneurotic edema, dermal mucinosis, lupus erythematosus, psoriasis, pustular subcorneal dermatosis, myxedematous lichen, and pyoderma gangrenosum have been reported [18]. Patients with cutaneous involvement showed a lower overall survival compared with those without cutaneous involvement [19].

Blue finger syndrome is not clearly described as a typical form of cutaneous presentation of MGUS, but there have been reports of blue digit syndrome in patients with MGUS in the absence of thromboembolic disease, at least evident in images, and in which no cause other than MGUS per se has been established [20].

Cutaneous manifestations in multiple myeloma are uncommon and have been classified as specific or nonspecific lesions. Cutaneous plasmacytoma is a specific but rare finding and easy to diagnose by histopathology. Other skin findings are common dermatosis, such as leukocytoclastic vasculitis, urticaria, autoimmune bullous diseases, and pyoderma gangrenosum [21].

When optimal treatment for multiple myeloma is promptly initiated, good prognosis with resolution of symptoms and organ dysfunction is commonly achieved. However, thalidomide administered in combination with multiagent chemotherapy and dexamethasone has been associated with an increased ischemic risk, which in our patient's case had never been provided [22]. Renal insufficiency accounts for most of the mortality, but complete recovery of renal function occurs frequently, even as soon as 60 days, as was seen in this case [23–25].

## 4. Conclusion

Although renal involvement and digital ischemia should always prompt investigations and treatment for vasculitis, its mimics must always be discarded before a diagnosis. Clinical awareness should be maintained during the whole process and, sometimes, there can be false test results. Monoclonal gammopathies should always be considered as a differential diagnosis for vasculitis with renal involvement and must be excluded, even in the absence of typical findings.

## Figures and Tables

**Table 1:** Test results of the approach.

Test performed	Test result	Normal range
Rheumatoid factor	8.6 UI/mL	0–12 UI/mL
Complement C3 levels	127 mg/dL	88–165 mg/dL
Complement C4 levels	34 mg/dL	14–44 mg/dL
Antinuclear antibodies	Negative	Negative
Anti-DNA antibodies	Negative	Negative
Extractable nuclear antigens: Ro, La, Sm, and RNP	0.2, 0.1, 0.1, and 0, respectively	0–0.9
C-ANCA	Negative	Negative
P-ANCA	Negative	Negative
HIV serology	Nonreactive	Nonreactive
Hepatitis B and C serology	Nonreactive	Nonreactive
Rapid plasma reagin	Nonreactive	Nonreactive
24-hour urine test	Creatinine clearance 42 mg/24 h	0.04–0.24 gr/24 h
	Proteins in urine 12 gr/24 h	
Albumin	4.2 gr/dL	3.5–5 gr/dL
Antiphospholipid antibodies: Anticardiolipin IgG and IgM, lupus anticoagulant, *β*2GP, IgG, and IgM	Negative	Negative
Cryoglobulins	Negative	Negative
Transthoracic echocardiography	There were no vegetations, cardiac tumor or other sources of emboli. Left ventricular ejection fraction 65%	—
Renal ultrasonography	No abnormalities	—
Thoracic X-ray	Normal	—
